# What Explains Education Disparities in Screening Mammography in the United States? A Comparison with The Netherlands

**DOI:** 10.3390/ijerph15091961

**Published:** 2018-09-08

**Authors:** Hale Koç, Owen O’Donnell, Tom Van Ourti

**Affiliations:** 1 Tinbergen Institute, 3062 PA Rotterdam, The Netherlands; hale.koc@gmail.com (H.K.); odonnell@ese.eur.nl (O.O.); 2 Department of Applied Economics, Erasmus School of Economics, 3062 PA Rotterdam, The Netherlands; 3 Department of Balkan, Slavic and Oriental Studies, University of Macedonia, 546 36 Thessaloniki, Greece

**Keywords:** breast cancer, screening mammogram, education gradient, screening program, health insurance

## Abstract

*Background:* In the U.S., less educated women are substantially less likely to receive screening mammography. It is not clear whether this is due to differences in access to screening or in perceptions of breast cancer risks and the effectiveness of screening. We weigh the plausibility of these two explanations by examining how the dependence of mammography on education changes after conditioning on indicators of access and perceptions. We also compare estimates for the U.S. with those for the Netherlands where there is universal access to a publicly financed screening program. *Method:* Cross-sectional and cross-country comparable individual level data from the American Life Panel (*n* = 646) and the Netherlands Longitudinal Internet Studies for the Social Sciences (*n* = 1398) were used to estimate and explain education disparities in screening mammograms given to American and Dutch women aged 40+. The education gradient was estimated using logit models. Controls were sequentially added to detect whether disparities were explained by differences in access or perceptions of risks and effectiveness. *Results:* In the United States, high school graduates were 11.5 percentage points (95% CI: 1–22 percentage points) less likely than college graduates to receive a screening mammogram in the previous two years. This education gradient was largely explained by differences in income, insurance coverage and receipt of medical advice. It was not explained by educational differences in the perceived risk of breast cancer and the effectiveness of mammography. There were no education disparities in receipt of mammography among Dutch women within the 50–75 age range covered by the national screening program. *Conclusion:* In the absence of a universal screening program in the U.S., determinants of access—income, insurance coverage and receipt of medical advice—appear to drive the education disparities in screening mammography.

## 1. Introduction

Breast cancer is the most commonly diagnosed cancer among women worldwide, accounting for 23% of all new cancer cases [1]. It is also the leading cause of cancer-attributable mortality of females around the globe being responsible for 14% of such deaths. Breast cancer is curable if detected sufficiently early and treated appropriately [2]. Screening mammography—a mammogram taken when there is no sign of breast cancer—is the recommended early detection tool for breast cancer due to its ability to detect pre-cancerous cells while they are still treatable [3].

While utilization of screening mammography increased steeply in the last two decades of the twentieth century [4], there remain large differences in uptake by education: In the United States [5,6] and a number of European countries [7,8,9], lower educated women tend to receive mammograms less often than their higher educated counterparts. This may reflect higher barriers to access faced by less educated women [9,10,11] but it could also arise from differences in perception of breast cancer prevalence, the incorporation of information available from objective risk factors and beliefs about the effectiveness of screening [5,9,10,12].

We use comparable data that include information on subjective perceptions of breast cancer risk and screening effectiveness to estimate and explain the education gradient in screening mammography in the United States and the Netherlands. Comparison of these two countries is instructive because they differ in access to screening. The Netherlands operates a universal, fully publicly subsidized screening program covering all women aged 50–75. Mammograms are delivered in mobile screening units to maximize accessibility [13,14] and all women in the eligible age range receive personalized invitations for a mammography screening via mail. There is no such program in the U.S. but the U.S. Preventive Services Task Force (USPSTF) recommends biennial screening of women aged 50–74 [3]. Since the 2010 Patient Protection and Affordable Care Act (PPACA), coverage of mammography screening without consumer cost-sharing is mandatory for new health plans, Medicare and Medicaid expansions. Despite these requirements, some American women, mostly those without insurance or in grand-fathered plans, still face out-of-pocket costs for mammograms [15]. Moreover and in contrast to Dutch women, American women in the recommended screening age range are not personally invited for a mammogram.

The absence of organized, subsidized screening, including universal invitations, in the United States leaves scope for socioeconomic differences in mammography uptake [5,6]. If these differences are mainly driven by financial and other access barriers, then they should become less evident after controlling for insurance coverage and receipt of doctor’s advice to get a mammogram. If, on the other hand, the education gradient is attributable mainly to perceptions of breast cancer risk and of the effectiveness of mammography, then it should be more sensitive to controlling for these factors. And these perceptions may influence the uptake of screening mammography in the Netherlands, where universal invitations for screening removes, or at least greatly lowers, the access barriers. We weigh the importance of differential access versus perceptions in generating the education gradient in screening mammography in the U.S. by examining how the gradient changes after conditioning on indicators of access and perceptions and by comparing the results with estimates for the Netherlands.

## 2. Methods

### 2.1. Data Sources

Data from two comparable internet panels were analysed—the American Life Panel (ALP), which is administered by the Rand Corporation and the Dutch Longitudinal Internet Studies for the Social Sciences (LISS), which is collected by CentERdata of Tilburg University [16,17,18]. ALP started in 2003, while LISS started in 2007. In both surveys, respondents complete questionnaires every month via the internet following a similar protocol. The probability sampling designs guarantee representativeness for the American and Dutch populations. Part of the interview time is reserved to generate repeated observations on the same variables for each panel member and the remaining time is used to collect data on topics that vary from wave to wave. We use the latter cross-sectional modules of ALP and LISS that include detailed individual-level information on breast cancer screening. Female respondents aged 40+, the earliest age recommended for screening [3,19], were extracted from the breast cancer module of the ALP (*n* = 646), which was fielded from mid-December 2011 until early January 2012 and from the disease prevention module of LISS (*n* = 1490), which was fielded in September 2008. The exact question wording of the crucial variables is further detailed in Section 2.2.1 and Section 2.2.2.

### 2.2. Variables

#### 2.2.1. Dependent Variables

The outcome of interest was receipt of a mammogram in the past 2 years. In LISS, the question was: “Have you had a mammogram in the last 2 years?” In ALP, respondents were asked “Have you ever had a screening mammogram” and if the answer was yes, “When did you have your most recent screening mammogram?” Based on these questions, the binary variable indicating mammogram usage in the last 2 years was constructed. The ALP explicitly asked about receipt of a screening mammogram, while no differentiation was made between receipt of a screening and a diagnostic mammogram in LISS, where the latter is conventionally defined as a test provoked by some indication of the possible presence of breast cancer. Therefore, Dutch women who reported previously having been diagnosed with breast cancer were dropped from the study sample.

#### 2.2.2. Independent Variables

Educational attainment was measured by the highest level completed with a diploma. To ensure comparability between the U.S. and the Netherlands, categories were constructed based on ISCED mappings of qualifications [20], distinguishing between low (upper secondary, that is, high school, education or less, ISCED < 4; reference category), middle (post-secondary non-tertiary education, ISCED = 4) and high education (higher education, ISCED > 4).

Some additional independent variables were common for the U.S. and the Netherlands, while others were country-specific. The set of common covariates included age [7,9] (5-year intervals for the Netherlands; 40–50, 50–65, 65–75 & 75+ for the U.S. to avoid small cell sizes), household income [7,21], race/ethnic origin [22,23] (binary indicators of Dutch and White respectively in the Netherlands and the U.S.), objective risk factors [19] (the number of first degree relatives with breast cancer for both the U.S. and the Netherlands coded into binary indicators for zero family members (reference category), 1 family member and more than one family member; and, for the Netherlands only, a binary indicator whether a women has given birth) and perceptions of breast cancer risk [5,12] and of mammogram effectiveness in reducing mortality from breast cancer. Breast cancer risk perception was measured by the reported probability of getting breast cancer within 5 years (number between 0 and 100). In LISS, the question was “What is the percent chance that you will get breast cancer in the next 5 years?”, while ALP uses “What do you think is the percent chance that you will get breast cancer in the next five years?” Perceived effectiveness of mammography in reducing the risk of death from breast cancer was measured on a four and five-point categorical scale for the U.S. and the Netherlands, respectively. In LISS it was derived from the relative difference between the stated probabilities obtained from the questions “What do you think is the chance that you will get breast cancer and die from it in the next 10 years? If you do have a mammogram every 2 years,” versus “If you do not have any mammograms.” In ALP, women answer the question “Imagine two women who are similar in many ways. They are the same age; they do the same activities; and, they eat similar foods. The first gets screening mammograms regularly and the second never gets screening mammograms. How much do you think the first woman reduces her chances of dying from breast cancer, compared to the second woman, who never gets screened?” In both countries, perceived effectiveness was transformed to a categorical variable which was entered as a set of binary indicators in our statistical analysis: expected mortality reductions lower than 25% were considered as low (reference category), reductions between 25% and 50% as medium low, reductions between 50% and 75% as medium high and reductions higher than 75% as high. Some Dutch women also revealed that mammograms may increase the probability of death from breast cancer (Negative category), while the option to state negative values for effectiveness was not given to women in ALP. Household income was measured before taxes for both countries and entered into the models as quartile group indicators (lowest income quartile is the reference category). 

Covariates included in the models estimated with U.S. data only were: (i) whether the respondent had full insurance (versus partial or no) coverage for a mammogram [22,23,24], (ii) whether a mammogram had been recommended by a health care provider [24,25] and (iii) perceived probabilities of a mammogram (number between 0 and 100) giving (a) a false positive, (b) a false negative and (c) a true positive. These perceived probabilities were derived from the questions “Imagine that you have an abnormal mammogram and a doctor recommends further testing to see whether or not you have breast cancer. What do you think is the percent chance that you do actually have breast cancer?”, “Imagine that you get a mammogram and it comes back with no signs of breast cancer. What do you think is the percent chance that you do actually have breast cancer?” and “Imagine that you have breast cancer that has not yet been detected and you go to your doctor for a routine screening mammogram. What do you think is the probability that the mammogram will detect the cancer?” For the Netherlands, an indicator of having received an invitation for a mammogram from the national breast cancer screening program [12] was included, as was having been invited in some other manner, which should mainly correspond to referrals given that Dutch women only very rarely receive a mammogram in a private clinic with no referral. Reporting having had a friend who died from breast cancer, which may influence risk perceptions, was also included in some models estimated for the Netherlands.

### 2.3. Statistical Analysis

We report descriptive statistics (percentages/means) of all dependent and independent analysis used in the analysis. The education gradient in mammography in the U.S. and the Netherlands was estimated by several logit models with education entered as a set of binary variables. The first model estimated included only age, in addition to education, to obtain the age-standardized education gradient. Additional covariates were cumulatively added to reveal the extent to which the association between education and screening was related to these variables. The second model added the objective risk factors, race/ethnic origin and subjective perceptions of breast cancer risk and mammogram effectiveness at detecting breast cancer and preventing death from it. The third model added income, insurance coverage and whether a health care provider had recommended getting a screening mammography in the case of the United States. For the Netherlands, whether an invitation for mammography had been received from the national screening program or through referral were added at this stage. 

From the logit model estimates, the difference between the probability of receiving a mammogram at a given education level and at the reference level was calculated for each observation and averaged across the sample. These average marginal effects (or adjusted risk differences) on the probability of getting a mammogram—and their associated 95% confidence intervals—were also calculated for the other covariates. Since the sample size of the ALP breast cancer module is less than half that of the LISS module and therefore power is lower in the former sample, we also provide the associated *p*-values of the two-sided significance tests. All analyses were done using Stata^®^ (StataCorp LLC, College Station, Texas, USA). Sample weights were applied throughout.

## 3. Results

### 3.1. Descriptive Statistics

Table 1 reports, for each country, the percentage of women within the recommended age range for screening (50–75) that received a mammogram and the percentage of women receiving a mammogram outside this range. At target ages, mammography prevalence in the previous two years was high (>80%) in both countries. At other ages, screening was still rather high in the United States but relatively uncommon in the Netherlands. In the United States, three-quarters of women aged 40+ were recommended by a health care provider to get a mammogram and the same fraction had insurance that fully covered the costs of regular mammograms. Neither fraction varied between women within and outside the recommended age range for screening. Less than 10% of Dutch women in the eligible age range reported not having been invited for screening by the national program. 

Table 2 presents means of the control variables by country. Around half of the American and Dutch women had no more than upper secondary (high school) education. American women had a higher expectation of getting breast cancer in the coming 5 years as well as a stronger belief in the effectiveness of a mammogram in preventing death from breast cancer.

### 3.2. Education Disparities in Screening Mammography 

Model 1 in Table 3 presents the age-adjusted differences by education in the probability of receiving a mammogram. There was a pronounced gradient in the United States: Women with low education were 11.5 percentage points less likely to be screened for breast cancer than their counterparts with higher education (*p*-value = 0.040). The middle education group is 10.4 points less likely to get a screening mammogram than the most educated (*p*-value = 0.056). There is no age-standardized education gradient in mammography use in the Netherlands (Wald test joint significance *p*-value = 0.397).

### 3.3. Accounting for Education Disparities 

The first two rows of Model 2 and 3 in Table 3 give the estimated education gradient in screening uptake after controlling for covariates in addition to age. Controlling for objective risk factors, race and perceptions of the risk of breast cancer and the effectiveness of mammography in averting death from this condition had little effect on the estimated education gradient in the United States (Model 2). The difference in the screening probability between the most and least educated women narrows slightly from 11.5 (Model 1) to 9.7 (Model 2) percentage points (*p*-value = 0.061). The estimated difference between the top two education groups actually widens from 10.5 to 12.5 points (*p*-value = 0.020). This persistence of the education gradient is not the result of differences in objective risk factors offsetting differences in risk perceptions. Controlling for age and objective risk factors only, the education gradient remains similar: low and middle educated women are estimated to have an 11.1 (*p*-value = 0.043) and 11.3 (*p*-value = 0.034) percentage point lower probability to be screened compared to higher educated women (not shown in Table 3).

Adding income, insurance coverage and medical advice to get screened (Model 3) has a much larger impact on the education gradient in the U.S., which decreases in magnitude (Wald test joint significance *p*-value = 0.653). Insurance coverage and medical advice are the strongest correlates of screening uptake. American women with insurance that fully covered mammography costs were 22.5 percentage points more likely to be screened than women with partial or no coverage; and those recommended by a medic to get screened were 38.4 percentage points more likely to do so. 

Controlling for objective risk factors, ethnicity and perceptions of risk and the effectiveness of screening did not change the conclusion that there is no education gradient in the age-standardized percentage of women receiving mammograms in the Netherlands (Wald test joint significance *p*-value = 0.580) (Table 3, Model 2). After controlling for income, being invited for screening from the national program or by referral, the least educated Dutch women had a 3.8 percentage point lower probability of getting a mammogram than the most educated women (*p*-value = 0.044). A program invitation itself raised the probability of getting a mammogram by 69 percentage points, while referral raised it by 42 points.

## 4. Discussion

There is a clear education disparity in age-standardized mammography receipt in the United States. Controlling for differences in perceptions of breast cancer risk and of screening effectiveness did not affect this gradient, while conditioning on income, insurance coverage and receipt of medical advice markedly weakened its magnitude. This suggests that lower educated American women were less likely to be screened because they were poorer, had less comprehensive insurance cover and, perhaps because of that, were less likely to come into contact with a physician who recommended mammography and not because they perceived less benefit from screening. The importance of financial barriers to mammography is evident from the fact that the education gradient vanishes (Wald test joint significance *p*-value = 0.268) after controlling only for income, in addition to age (estimates not shown in Table 3). Less educated, poorer women do not have the insurance cover and access to medical advice that are the main determinants of mammography screening in the United States. 

In the Netherlands, a moderate education gradient in mammography emerges only after controlling for differences in the propensity to be invited for screening through the national program or by referral. Further analysis revealed that this education disparity existed among women who were not invited for screening by the national program. Among such women, who are uninvited mostly because they are outside the 50–75 age range, the least educated were 5.6 percentage points (*p*-value < 0.027) less likely than the most educated to get a mammogram (estimates not shown in Table 3). Given the lower incidence of life-threatening breast cancer in this age range and risks arising from false positive screens, it is not obvious that this disparity is to the advantage of the more educated.

### 4.1. Role of Health Insurance and Access to Screening

In the U.S., the strongest correlates of screening uptake were medical advice and insurance coverage (Table 3, Model 3). In the Netherlands, it was an invitation for screening (Table 3, column 3). These determinants are largely responsible for the differences between the two countries in the distribution of breast cancer screening. Table 4, column ‘Medical advice to get mammogram’ shows that, in the U.S., the probability of receiving medical advice to get a mammogram did not vary by age (Wald test joint significance *p*-value = 0.981) and by income (Wald test joint significance *p*-value = 0.949). It varied (only marginally) with objective risk factors (Wald test joint significance *p*-value = 0.218) and by education (Wald test joint significance *p*-value = 0.161) and, most strongly, by insurance coverage (*p*-value = 0.001). In turn, comprehensive insurance coverage of mammogram was determined by age (Wald test joint significance *p*-value = 0.006), due to Medicare qualification at 65 but also by income (Wald test joint significance *p*-value = 0.030) and education (Wald test joint significance *p*-value = 0.138) but not by objective risk factors (joint significance *p*-value = 0.289) (column ‘Insurance coverage for mammogram’).

Higher educated and better-off American women are more likely to be insured, which is associated with a greater likelihood of being advised to undertake a mammogram, perhaps because doctors are more likely to recommend screening to patients they believe can afford it [25,26]. Better-off women may also be more likely to consult with doctors known to recommend screening, or may be more successful in asking for a referral.

In the Netherlands, invitation for screening, which is the dominant determinant of mammography receipt, was almost exclusively determined by age and objective risk factors and not by education or income (Table 4, columns ‘Invited for mammogram by national screening program’ and ‘… through referral’). Differences in the role of financial barriers and in access to medical advice seem important in explaining the strong socioeconomic gradient in breast cancer screening in the U.S. and its absence in the Netherlands.

### 4.2. Strengths and Weaknesses

This study is strengthened by the availability of comparable data on perceptions of breast cancer risk and mammogram effectiveness allowing consideration of the hypothesis that education disparities in screening behaviour reflect differences in ability to accumulate and process information. Inevitably, indicators of perceived breast cancer risk and mammogram effectiveness are self-reported making these indicators prone to self-reporting errors. This is also true of the other variables used in this study.

A weakness of the study is that while the sample sizes are sufficient for the main analyses, the U.S. sample does limit the extent of disaggregated analysis that is feasible and, together with the categorical nature of the income information, is partly responsible for the insignificance of income despite its importance in reducing the education gradient.

While we cannot exclude the possibility of time-related confounding due to the breast-cancer modules of LISS and ALP referring to different years (2008 and 2011–2012); its impact is most likely limited as the take-up of mammography screening has been very stable in the Netherlands between the start of the screening program and 2011–2012 [27], the years when the questionnaire was fielded in ALP. Moreover, using U.S. data post-PPACA allows unravelling disparities after mammogram coverage became compulsory under Medicare and new health plans.

While insurance coverage is identified as an important correlate of breast cancer screening in the U.S., it is not possible to conclude from the analysis that insurance has a causal positive impact on the likelihood of receiving a mammogram. It could be that women who want to be screened are more likely to purchase an insurance plan.

Control for some objective risks increases the likelihood that the education gradient in insurance coverage in the U.S. implies differential financial barriers to screening for equal risk. While the most important risk factors such as age and the number of relatives dying of breast cancer have been included, we cannot exclude remaining confounding because of lack of control for less important risk factors, such as nulliparity and later age at first birth, which are more common among higher educated women [28,29].

## 5. Conclusions

There is a clear socioeconomic gradient in breast cancer screening in the U.S. that appears to reflect differences in financial barriers and insurance coverage. Differences in perceptions of breast cancer risks and screening effectiveness appear to be less important in explaining education disparities. This, together with absence of a gradient in the Netherlands, where there is a universal breast cancer screening program and risk perceptions are also unimportant in explaining screening uptake, suggests that access, rather than information and promotion programs may be the more important determinant of the distribution of mammography.

## Figures and Tables

**Table 1:** Percentage of women receiving screening mammograms by country and age.

	United States		The Netherlands	
	Age		Age	
	50–75	40–50, 75+	50–75	40–50, 75+
Mammogram in last 2 years	80.82	69.38	84.90	16.39
Mammogram				
recommended by doctor	76.00	74.60	n.a.	n.a.
covered by insurance	75.95	74.80	n.a.	n.a.
invite from screening program	n.a.	n.a.	91.41 ^a^	5.03 ^b^
invite through referral	n.a.	n.a.	3.60	8.57
Sample size (*n*)	501	145	861	537

Notes: 50–75 years is the recommended age range for screening mammography in the U.S. and the range covered by the universal screening program in the Netherlands; ^a^: The remaining 8.59% includes women who left the program by their own choice and refused to receive further invitations and women who had recently entered the eligible age range at the time of interview but had not yet received an invitation for screening; ^b^: Women aged 75–77 might have been screened during the last two years when they were still below 75 after receiving an invitation from the national program. All numbers are percentages; n.a.: not available.

**Table 2:** Means of control variables by country, women aged 40+.

	United States	The Netherlands
Age group		
40–44 (U.S. 40–49) ^a^	30.07	16.74
45–49		18.60
50–54 (U.S. 50–64)	42.00	16.52
55–59		16.24
60–64		13.52
65–69 (U.S. 65–75)	20.24	9.80
70–75		5.51
75+	7.69	3.08
Education		
Low (ISCED < 4)	47.00	53.72
Middle (ISCED = 4)	20.54	19.60
High (ISCED > 4)	32.46	26.68
Breast cancer in the family		
No	85.03	87.98
Yes—1 family member	11.46	10.80
Yes—1+ family member	3.51	1.22
Has given birth	n.a.	84.26
Race/Ethnic origin		
Dutch	n.a.	90.70
White	77.11	n.a.
Having a friend who died of breast cancer	n.a.	48.71
Perceived risk of getting breast cancer in the next 5 years	24.64	20.28
Perceived effectiveness of mammogram at preventing death		
Negative	n.a.	12.45
Low	20.66	24.96
Medium-low	22.34	23.03
Medium-high	32.05	21.67
High	24.94	17.88
Perceived probability of mammogram:		
giving a false-positive result	32.78	n.a.
giving a false-negative result	13.40	n.a.
detecting breast cancer	62.22	n.a.
Gross household income ^b^		
Low	30.06	25.75
Low-middle	22.87	24.25
High-middle	27.98	25.04
High	19.10	24.96
Sample size (*N*)	646	1398

^a^ Wider age intervals are used for the U.S. because of the smaller sample size; ^b^ The income quartiles constructed from the U.S. data did not divide the sample into four equally sized groups since they were derived from a categorical income variable; n.a.: not available. All numbers are percentages.

**Table 3:** Age-adjusted differences in probability of screening mammogram by country.

	United States			The Netherlands		
	Model 1	Model 2	Model 3	Model 1	Model 2	Model 3
Education (ref = High)						
Low	−0.115 ** (−0.224; −0.005)	−0.097 * (−0.199; 0.004)	0.024 (−0.065; 0.113)	−0.027 (−0.072; 0.018)	−0.023 (−0.069; 0.023)	−0.038 ** (−0.075; −0.001)
Middle	−0.104 * (−0.210; 0.003)	−0.125 ** (−0.229; −0.020)	−0.011 (−0.094; 0.073)	0.001 (−0.054; 0.056)	−0.004 (−0.059; 0.052)	−0.025 (−0.070; 0.021)
Breast cancer in the family (ref = No)						
Yes—1 family member		0.057 (−0.044; 0.159)	0.051 (−0.042; 0.143)		0.054 * (−0.009; 0.117)	0.045 * (−0.004; 0.094)
Yes—1+ family member		−0.006 (−0.289; 0.277)	−0.010 (−0.166; 0.146)		0.104 (−0.100; 0.308)	−0.002 (−0.157; 0.154)
Has given birth					0.009 (−0.044; 0.063)	0.028 (−0.017; 0.073)
Race/Ethnic origin						
Dutch		n.a.	n.a.		−0.008 (−0.073; 0.056)	−0.010 (−0.061; 0.041)
White		−0.135 ** (−0.253; −0.017)	−0.075 (−0.181; 0.031)		n.a.	n.a.
Having a friend who died of breast cancer		n.a.	n.a.		0.042 **(0.003; 0.081)	0.021 (−0.011; 0.052)
Perceived risk of getting breast cancer in the next 5 years		0.001 (−0.001; 0.003)	0.000 (−0.001; 0.002)		0.001 ** (0.000; 0.002)	0.001 ** (0.000; 0.002)
Perceived effectiveness mammo preventing death (ref = Low)						
Negative		n.a.	n.a.		−0.018 (−0.085; 0.049)	0.020 (−0.035; 0.074)
Medium-low		0.088 (−0.098; 0.275)	−0.001 (−0.123; 0.121)		0.019 (−0.036; 0.074)	0.039 * (−0.006; 0.083)
Medium-high		0.300 *** (0.146; 0.454)	0.166 *** (0.060; 0.273)		0.077 *** (0.022; 0.132)	0.054 ** (0.010; 0.099)
High		0.235 *** (0.067; 0.403)	0.081 (−0.032; 0.192)		0.048 (−0.011; 0.107)	0.040 * (−0.006; 0.087)
Perceived probability of mammogram						
giving a false-positive result		0.001 (−0.002; 0.004)	0.000 (−0.002; 0.002)		n.a.	n.a.
giving a false-negative result		−0.001 (−0.004; 0.003)	0.000 (−0.002; 0.003)		n.a.	n.a.
detecting breast cancer		0.000 (−0.002; 0.001)	0.001 * (0.000; 0.002)		n.a.	n.a.
Income (ref = Low)						
Low-middle			−0.028 (−0.136; 0.081)		0.013 (−0.030; 0.055)	
Middle-high			0.006 (−0.089; 0.102)		0.009 (−0.036; 0.054)	
High			0.072 (−0.043; 0.186)		0.029 (−0.017; 0.074)	
Mammogram						
covered by insurance			0.225 *** (0.127; 0.323)			n.a.
recommended by doctor			0.384 *** (0.260; 0.508)			n.a.
invitation by program			n.a.			0.693 *** (0.613; 0.773)
invitation in other manner			n.a.			0.415 *** (0.378; 0.452)
Sample size (*N*)	646			1398		

Notes: Average marginal effects from logit models are reported. All models control for age. For each individual, we calculate the marginal effect of a covariate category by taking the difference between the predicted probability of screening in that category and in the reference category. Average marginal effect is the average of individual marginal effects. * *p* < 0.1, ** *p* < 0.05, *** *p* < 0.01. 95% confidence intervals are given in parentheses. n.a.: not available.

**Table 4:** Differences in probability of medical advice, insurance coverage and screening invitation for mammogram.

	United States		The Netherlands	
	Medical Advice to Get Mammogram	Insurance Coverage for Mammogram	Invited for Mammogram by National Screening Program	Invited for Mammogram through Referral
Age group (ref = 40–49)				
50–64	0.022 (−0.093; 0.138)	−0.023 (−0.148; 0.103)	0.864 *** (0.837; 0.891)	−0.069 *** (−0.102; −0.036)
65–75	0.016 (−0.123; 0.155)	0.179 ** (0.043; 0.316)	0.859 *** (0.817; 0.901)	−0.082 *** (−0.118; −0.046)
75+	0.030 (−0.163; 0.223)	0.243 *** (0.107; 0.380)	0.129 ** (0.012; 0.246)	n.a. ^a^
Education (ref = High)				
Low	−0.038 (−0.146; 0.070)	−0.107 * (−0.219; 0.005)	0.019 (−0.013; 0.052)	0.004 (−0.026; 0.035)
Middle	−0.115 * (−0.235; 0.006)	−0.016 (−0.104; 0.072)	0.015 (−0.026; 0.056)	−0.006 (−0.039; 0.027)
Breast cancer in the family (ref = No)				
Yes—1 family member	0.090 * (−0.003; 0.183)	0.087 (−0.020; 0.195)	−0.028 (−0.069; 0.012)	0.088 *** (0.030; 0.147)
Yes—1+ family member	−0.011 (−0.260; 0.239)	0.102 (−0.112; 0.315)	−0.066 (−0.192; 0.061)	0.342 *** (0.106; 0.578)
Has given birth	n.a.	n.a.	−0.006 (−0.042; 0.029)	−0.002 (−0.035; 0.032)
Race/ethnic origin				
Dutch	n.a.	n.a.	−0.009 (−0.053; 0.034)	0.006 (−0.032; 0.045)
White	−0.129 ** (−0.249; −0.009)	−0.016 (−0.149; 0.117)	n.a.	n.a.
Income (ref = Low)				
Low-middle	0.016 (−0.111; 0.144)	−0.085 (−0.242; 0.072)	0.015 (−0.020; 0.050)	−0.036 * (−0.074; 0.003)
Middle-high	0.031 (−0.083; 0.146)	0.065 (−0.070; 0.200)	0.035 ** (0.000; 0.071)	−0.019 (−0.058; 0.021)
High	0.002 (−0.157; 0.160)	0.130 * (−0.002; 0.262)	0.003 (−0.036; 0.042)	−0.033 (−0.072; 0.006)
Invitation in other manner	n.a.	n.a.	−0.405 *** (−0.510; −0.300)	n.a.
Covered by insurance	0.204 *** (0.085; 0.323)	n.a.	n.a.	n.a.
Sample size (*N*)	646		1398	

Notes: Average marginal effects from logit models are reported. For each individual, we calculate the marginal effect of a covariate category by taking the difference between the predicted probability of screening in that category and in the reference category. Average marginal effect is the average of individual marginal effects. * *p* < 0.1, ** *p* < 0.05, *** *p* < 0.01. 95% confidence intervals are given in parentheses. n.a.: not available. ^a^: there are no women in the 75+ age range that have been invited in another manner.
